# Development of Photo-Activated ROS-Responsive Nanoplatform as a Dual-Functional Drug Carrier in Combinational Chemo-Photodynamic Therapy

**DOI:** 10.3389/fchem.2018.00647

**Published:** 2019-01-09

**Authors:** Yu-Cheng Chang, Andrea C. del Valle, Huan-Pu Yeh, Yue He, Yu-Fen Huang

**Affiliations:** ^1^Department of Biomedical Engineering and Environmental Sciences, National Tsing Hua University, Hsinchu, Taiwan; ^2^Laboratory of Quality & Safety Risk Assessment for Citrus Products, Ministry of Agriculture, Citrus Research Institute, Southwest University, Chongqing, China

**Keywords:** combined therapy, photodynamic thearpy, chemotherapy, ROS-responsive, nanocarrier

## Abstract

Dual functional drug carrier has been a modern strategy in cancer therapy because it is a platform to elicit additive and synergistic effects through combination therapy. Photo-activated external stimuli such as reactive oxygen species (ROS) also ensure adequate drug delivery in a precise temporal and spatial manner. However, current ROS-responsive drug delivery systems usually require tedious synthetic procedures. A facile one-pot approach has been reported herein, to obtain self-assembled polymeric nanocarriers (NCs) for simultaneous paclitaxel (PTX)- and Rose Bengal (RB)-loading to achieve combined chemo-photodynamic therapy and controlled drug release in responsive to a light-induced ROS stimulus. To encapsulate these hydrophobic and hydrophilic drugs, chitosan (CTS), branched polyethylenimine (*b*PEI) and polyvinyl alcohol (PVA) were selected and fabricated into nanoblended matrices through an oil-in-water emulsion method. The amphiphilic properties of CTS permit simultaneous entrapment of PTX and RB, while the encapsulation efficiency of RB was further improved by increasing the amount of short-chain *b*PEI. During the one-step assembly process, bovine serum albumin (BSA) was also added to condense the cationic tripolymer mixtures into more stable nanocarriers (BNCs). Hyaluronic acid (HA) was subsequently grafted onto the surface of BNCs through electrostatic interaction, leading to the formation of HA-BSA/CTS/PVA/*b*PEI-blended nanocarriers (HBNCs) to achieve an efficient prostate-cancer-cell uptake. Importantly, in response to external light irradiation, HBNCs become destabilized owing to the RB-mediated photodynamic action. It allows an on-demand dual-payload release to evoke a simultaneous photodynamic and chemo treatment for cancer cell eradication. Thus, HBNCs present a new promising approach that exhibits a specific vulnerability to RB-induced photosensitization. The consequent dual-cargo release is also expected to successfully combat cancer through a synergistic anti-tumor effect.

## Introduction

As an extremely complex disease, cancer causes a serious threat to human health. It involves numerous tempo-spatial changes in cell physiology along with complex signaling pathways, enabling tumor cells to evade programmed cell death, thus making the treatment extremely challenging (Hanahan and Weinberg, 2000, 2011). Despite the fact that a multitude of promising therapeutic strategies has been developed, cancer remains a major cause of morbidity and mortality, as well as the top public health problem worldwide (Kanavos, 2006; Gellad and Provenzale, 2010; Siesling et al., 2015; Hopkins and Secrest, 2018). Chemotherapy, as an orthodox antitumor option in clinic (Chabner and Roberts, 2005; DeVita and Chu, 2008), is however, limited by its serious side effects, poor water-solubility of chemotherapeutic drugs, and intricate multidrug resistance, leading to undesired therapeutic outcomes in cancer treatment (Luqmani, 2005; Szakacs et al., 2006). To address this puzzle, combining chemotherapy with other different forms of treatments has become a promising strategy (Peng et al., 2008; Zhang et al., 2011, 2016; Wang et al., 2013).

Photodynamic therapy (PDT) (Dougherty et al., 1998; Nseyo et al., 1998), a popular, non-invasive cancer treatment, which relies on photosensitizing agents, O_2_ and light activation to produce reactive oxygen species (ROS) for destructing cellular components and tumor vasculature, has attracted increasingly attention for combination therapy with chemotherapeutic drugs (Peng et al., 2008; Zhang et al., 2016). In recent years, nanomaterials such as polymer (Peng et al., 2008), liposomes (Ma et al., 2018), metal nanoparticles (Shiao et al., 2014) and hydrogels (Xu et al., 2017) have been considered as potential co-drug delivery vesicles for the simultaneous encapsulation of photosensitizer and chemo drug to realize combination therapy. This co-delivery system not only can lead to additive or synergistic drug interactions to confer a beneficial effect on treatment response, but also can deliver the therapeutic payloads in a spatiotemporally controlled manner in response to the specific endogenous or exogenous stimuli, while protecting premature drug leakage, degradation, or modification in the biological environment. Photoinduced ROS generation, for instance, has been considered as one potential trigger to achieve desired payload release profiles. Many polymer-based nanoparticles are composed of ROS-sensitive segments, which are readily oxidized and disintegrated in ROS-abundant conditions, making them ideal materials for oxidation-dependent drug release (Napoli et al., 2004; Gupta et al., 2012; Yue et al., 2016; Wei et al., 2018). Yue et al. developed a ROS-responsive polymer-based nanoparticle for applications in dual-functional drug delivery (Yue et al., 2016). A block copolymer comprised of triphenylphosphonium and polyethylene glycol (PEG) which was functionalized with thioketal linker-modified camptothecin, can encapsulate the photosensitizer Zinc phthalocyanine by blending the block copolymer with 1, 2-distearoyl-sn-glycero-3-phosphoethanolamine-N-[methoxy (polyethylene glycol)]. The thioketal linker is ROS-responsive and camptothecin can be released upon ROS cleavage, thus successfully realizing combinational chemo-photodynamic therapy. Wei et al. developed another ROS-responsive nanoplatform based on a protoporphyrin-conjugated and dual chemotherapeutics-loaded polymer micelle (Wei et al., 2018). This polymer comprised acetylated-chondroitin sulfate (AC-CS) as the hydrophilic block and protoporphyrin grafted on AC-CS via an ester bond as the hydrophobic block. Under a red light irradiation, ROS generation from protoporphyrin disassociated this polymer, allowing great improvement in therapeutic effect *via* the combination of chemotherapy and PDT.

**Figure 1:**
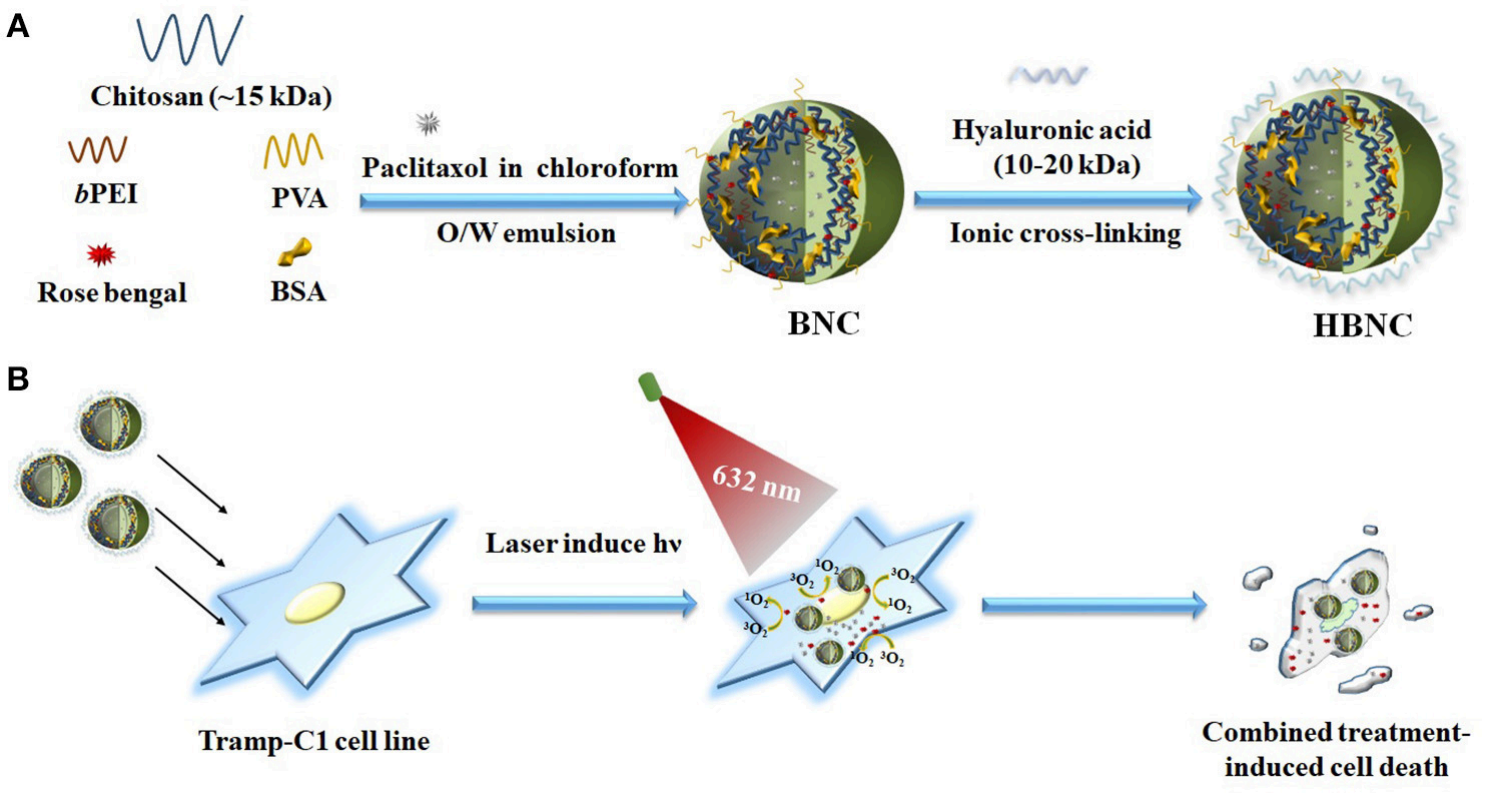
Schematic illustration of fabrication of ROS-responsive polymeric nanocarriers for co-delivery of two different drugs (RB and PTX) through an oil-in-water emulsion method **(A)** and the possible mechanism in targeted PDT and chemotherapy to prostate cancer Tramp-C1 cells **(B)**.

Although promising, these polymer nanoparticles mentioned above usually require complicated synthetic routes to end up with desirable covalent linkers showing sufficient ROS-responsiveness. An ionically physical cross-linked network that is suitable for an efficient encapsulation of multiple drugs and subsequent cargo release activated by ROS is consequently highly demanded in cancer therapy. Based on the concept outlined in our recent work (Yeh et al., 2018), branched polyethylenimine (*b*PEI) can act as a photoinducible switch for effective cargo trapping and disposal in response to a photooxidation process sensitized by Rose Bengal (RB). A dual-functional stimuli-responsive nanocarrier was therefore designed herein, and obtained by ionically cross-linking of a self-assembled, amphiphilic polymeric network. We expect the entangled coacervate generated in this study, can potentiate the therapeutic efficacy via a concurrent treatment of PDT and chemotherapy. As depicted in Figure 1, a one-step oil-in-water emulsion-solvent evaporation method was utilized for nanoparticle fabrication. Paclitaxel (PTX), as a hydrophobic chemotherapeutic agent was dissolved in an oil phase (chloroform) and dispersed into an aqueous phase containing chitosan (CTS), polyvinyl alcohol (PVA), low molecular weight *b*PEI (1.8 kDa) as well as bovine serum albumin (BSA) and RB molecules, followed by emulsification. After the introduction of hyaluronic acid (HA), as a CD44-targeting agent (Peach et al., 1993), the gradual removal of the residual organic solvent by evaporation led to the formation of HA-BSA/CTS/PVA/bPEI-blended nanocarriers (HBNCs). The amphiphilic properties of the cationic tripolymer nanocarriers (NCs) permit effective entrapment of both PTX and negatively charged RB molecules. A more rigid polymeric matrix (BNCs) was also obtained with the inclusion of BSA during the single-pot non-covalent interaction. HA was electrostatically attached onto the BNCs surface, endowing the resultant HBNCs with prostate cancer-targeting capability. When irradiated with the external light, RB-induced ROS generation is expected to diminish the strength of ionic cross-links of HBNCs (Yeh et al., 2018), leading to the simultaneous release of the dual drugs. The dual-functional co-delivery platform can yield a synergistic therapeutic effect by the combination of PDT and chemo-cytotoxicity to improve the therapeutic efficacy in tumor cells.

## Experimental Section

### Chemicals

Poly(vinyl alcohol) (PVA) and branched polyethylenimine (*b*PEI) were purchased from Sigma-Aldrich (St. Louis, MO, USA) and Alfa Aesar (Ward Hill, MA, USA) with Mw of 9,000~10,000 and 1,800 Da, respectively. The low molecular weight of chitosan was purchased from Polyscience, Inc (Warrington, PA, USA). Bovine serum albumin (BSA) was purchased from Sigma-Aldrich (St. Louis, MO, USA). Chloroform and acetonitrile were purchased from Merck Schuchardt (Hohenbrunn, Germany) and J.T. Baker (Center Valley, PA, USA), respectively. 2′,7′-Dichlorofluorescin diacetate (DCFH-DA), and 4,5,6,7-Tetrachloro-2′,4′,5′,7′-tetraiodofluorescein disodium salt (Rose Bengal) were purchased from Sigma-Aldrich (St. Louis, MO, USA). Paclitaxel was purchased from Seedchem (Vic Melbourne, Australia). Dried sodium hyaluronate (HA) (10–20 K Da) was purchased from Lifecore (Center Valley, PA, USA). Transferrin from human serum and 4',6-diamidino-2-phenylindole (DAPI) were purchased from Invitrogen (Carlsbad, CA, USA). DMEM, fetal bovine serum, and trypan blue were purchased from Grand Island, NY, USA. AlamarBlue was purchased from AbD Serotec (Oxford, OX5 1GE, UK). Sodium chloride, calcium chloride, magnesium chloride, potassium chloride, monopotassium phosphate, sodium phosphate dibasic, L-glucose were purchased from J.T.Baker (Center Valley, PA, USA). Dulbecco's phosphate-buffered saline (DPBS) was purchased from Biosource (Camarillo, CA, USA). Deionized water (18.2 M Ω cm) was used to prepare all of the aqueous solutions. For the cellular experiments, all of the reagents, buffers and culture medium were sterilized by steam autoclave (121°C, 40 min) or filtration (0.22 μm pore size, Millipore), and maintained under a sterile condition.

### Cell Lines and Buffers

Tramp-C1 (transgenic adenocarcinoma of the mouse prostate), was obtained from American Type Culture Collection (ATCC, Manassas, VA, USA). Cells were cultured in suspension in DMEM medium supplemented with 10% FBS and 1% penicillin-streptomycin (Invitrogen, Carlsbad, CA, USA) at 37°C in a balanced air humidified incubator with an atmosphere of 5% CO_2_. The cells were passaged every 2–3 days. Cell density for every experimental assay was determined using a hemocytometer; purity of cell density was determined by visual microscopic inspection of the nuclei stained by trypan blue.

### Synthesis of CTS/PVA/*b*PEI-Blended Nanocarriers (NCs), HA-CTS/PVA/*b*PEI-Blended Nanocarriers (HNCs), BSA/CTS/PVA/*b*PEI-Blended Nanocarriers (BNCs) and HA-BSA/CTS/PVA/*b*PEI-Blended Nanocarriers (HBNCs)

CTS solutions of 5 mg/mL were prepared by dissolving chitosan in 0.5% aqueous acetic acid solution at room temperature with vortexing. PVA (10 k Da), *b*PEI (800 Da) and BSA were dissolved in water, respectively. NCs, HNCs, BNCs, and HBNCs were produced by emulsion/solvent evaporation method. The weight of CTS, PVA, *b*PEI, BSA and HA in different NCs were listed in Table S1. Chloroform (1 mL) with PTX was added into a various aqueous solution (4 mL) containing different polymeric mixtures. To form oil-in-water (O/W) emulsion, ultrasonication was then applied to the solution with an ice bath for 10 min using a tip-type Qsonica sonicator (pulse mode with on: 10 sec, off 10 sec, and output of 60). To prepare HNCs and HBNCs, HA solution was added into the responsive solution drop by drop accompany with vortexing (250 rpm/s) until CHCl_3_ was completely evaporated. After solvent evaporation, the remaining polymer and solvent were removed by centrifugation (40,000 g, 20 min) and washed by phosphate buffer (PB, 10 mM) twice to obtain the purified polymeric NCs, HNCs, BNCs, and HBNCs.

### Synthesis of HBNCs With Different Ratio of Polymer

HBNCs were produced by emulsion/solvent evaporation method. The weight ratios of CTS, PVA, *b*PEI and HA to BSA in different samples were listed in Table 1. The preparation and purification processes were the same with above.

**Table 1:** Characterization of HBNCs with different constitutes in H_2_O.

**Sample**	**CTS (mg/mL)**	**PVA (mg/mL)**	**PEI (mg/mL)**	**BSA (mg/mL)**	**HA (mg/mL)**	**Size (d.nm)**	**Zeta (mv)**	**PDI**	**EE_**RB (%)**_**
(a)	1	0.4	0.4	0.1	0.2	220 ± 14	17.1 ± 2.5	0.18 ± 0.14	60.7 ± 2.7
(b)	0	0.4	1.0	0.1	0.2	246 ± 41	1.8 ± 1.3	0.57 ± 0.06	23.2 ± 4.2
(c)	1	0.04	0.4	0.1	0.2	870 ± 114	18.2 ± 0.6	0.43 ± 0.64	n.d.
(d)	1	0.4	0	0.1	0.2	238 ± 26	26.3 ± 1.4	0.38 ± 0.01	33.1 ± 0.3
(e)	1	0.4	0.4	0.1	0.4	516 ± 112	14.7 ± 0.4	0.31 ± 0.09	n.d.

*EE, encapsulation efficiency; n.d., not determined*.

### Preparation of Paclitaxel (PTX) and Rose Bengal (RB)-Loaded Polymeric HBNCs

To fabricate PTX/RB incorporated nanoparticles, as shown in Scheme S1, RB (40 μM) was added to the aqueous polymer blend solution (4 mL), followed by the addition of 1 mL chloroform with 100 μM PTX. Then adopt the same synthetic strategy described above to form dual drugs-loaded polymeric HBNCs.

### Characterization of the Physiochemical Properties of Polymeric NCs, HNCs, BNCs, and HBNCs

The hydrodynamic diameter and zeta potential values of the constructed polymeric NCs, HNCs, BNCs, and HBNCs were measured by a dynamic light scattering (DLS) instrument (Malvern Instruments, United Kingdom), respectively. The morphology and size were also confirmed by transmission electron microscopy (Hitachi, Tokyo, Japan).

### Characterization of the Stability of NCs, HNCs, BNCs, and HBNCs in a Different Environment

NCs, HNCs, BNCs, and HBNCs were dissolved in DPBS and DMEM/FBS (10%), respectively. Make these nanoparticles to equilibrate with the environment for 1 h. The hydrodynamic diameter and zeta potential values of these constructed polymeric nanoparticles were measured by a DLS, respectively.

### Characterization of Drugs Loading

The loading efficiency of RB into NCs, HNCs, BNCs, and HBNCs were determined by UV-Vis absorption spectrum measurement (Cary 100, Varian, Palo Alto, CA, USA), respectively. The absorption spectrum of the supernatant, substrate, and the original solution were measured to calculate the loading efficiency. The loading amount of PTX was quantified by high-performance liquid chromatography analysis (HPLC, Eclipse XDB-C18) using an eluent of H_2_O/Acetonitrile (1:1) at 1.0 mL/min.

### Characterization of Stability of Dual Drugs-Loaded HBNCs in a Different Environment

RB/PTX-loaded HBNCs dispersed in DPBS containing 10% FBS was transferred to dialysis vials (3500 Da cutoff; Slide-A-Lyzer™ MINI Dialysis Devices) and dialyzed against DPBS at ambient temperature. The amount of drug remaining inside the dialysis tubing was quantified using a calibration curve at selected time intervals.

### ROS Assay

ROS production of RB-loaded polymeric HBNCs and RB/PTX-loaded polymeric HBNCs were measured using the fluorescence probe DCFH-DA, respectively. 1 μL of DCFH-DA (1 mM) was added to RB-loaded HBNCs solution (200 μL) and RB/PTX-loaded HBNCs solution (200 μL) prior to light exposure (632 nm, 15 mW/cm^2^) for 30, 60 and 120 min, respectively. The increase in fluorescence signal as a consequence of ROS generation was acquired using Fluorescence spectrophotometer at an excitation wavelength of 488 nm and an emission wavelength of 530 nm. Concurrently, ROS production by free RB (20 μM) was measured under the same condition as the control.

Intracellular ROS were further detected by flow cytometry using DCFH-DA. Briefly, Tramp-C1 cells were seeded at a density of 3 × 10^4^ cells per 48-well plate for 12 h attachment. Cells were then incubated with RB, HBNCs, PTX-HBNCs, RB-HBNCs, and RB/PTX-HBNCs in complete culture medium for 6 h and washed twice in DPBS, respectively. Five milli meter DCFH-DA prepared in DPBS was added to the cells for 15 min at 37°C. Following 1 h red light exposure, cells were trypsinized and collected in the tube by centrifugation (1,000 g for 5 min) and resuspended in 200 μL washing buffer [4.5 g/L glucose and 5 mM MgCl_2_ in DPBS] for flow cytometry analysis (excitation = 488 nm; emission = 530 nm). For each analysis, at least 10,000 events were counted.

### Release of Drugs From Polymeric HBNCs Upon Light Irradiation

The release study was conducted as follows: RB/PTX-loaded HBNCs was dispersed in DPBS with 10% fetal bovine serum (pH 7.4) at 37°C. The complex solution (200 μL) was transferred to dialysis vials (3500 Da cutoff; Slide-A-Lyzer™ MINI Dialysis Devices) and dialyzed against DPBS at ambient temperature. After red light or green laser light (15 mW/cm^2^) illumination for various periods, buffer solution outside the dialysis vials was then taken for measurement at selected time intervals. The amount of RB release was quantified from the respective calibration curves. The release amount of PTX was quantified by HPLC.

### Intracellular Uptake of RB/PTX-Loaded Polymeric HBNCs

To observe the cellular uptake of RB/PTX-loaded HBNCs, Tramp-C1 cells were seeded at a density of 2 × 10^4^ cells on 10 × 10 mm sterile cover glasses inserted into 48-well plates for 12 h attachment. Cells were then incubated with RB/PTX-loaded HBNCs in complete culture medium for 6 h and washed twice in DPBS. For microscopic imaging, cells were fixed with 4% paraformaldehyde for 10 min and monitored by confocal laser scanning microscopy (C2 plus Confocal system, Nikon, Tokyo, Japan). Endosomes were stained with transferrin, Alexa Fluor 633 conjugate (200 nM) for 30 min. Nuclei were stained with 4',6-diamidino-2-phenylindole (DAPI, 1.0 μM) for 15 min.

### Binding Affinity Analyses

Briefly, Tramp-C1 cells were seeded at a density of 3 × 10^4^ cells per 48-well plate for 12 h attachment. Cells were then incubated with RB/PTX-BNCs and RB/PTX-HBNCs in complete culture medium for 6 h and washed twice in DPBS, respectively. Cells were trypsinized and collected in the tube by centrifugation (1,000 g for 5 min) and resuspended in 200 μL washing buffer for flow cytometry analysis (excitation = 488 nm; emission = 530 nm). For each analysis, at least 10,000 events were counted.

### Cytotoxicity Assay

Tramp-C1 cells were seeded at a density of 5 × 10^3^ cells per 96-well plate for 12 h. Cells were washed once and then incubated with HBNCs, RB-HBNCs, PTX-HBNCs, and RB/PTX-HBNCs in complete culture medium for 6 h and washed twice in DPBS, respectively. In PDT studies, treated cells were exposed to a red light (15 mW/cm^2^) for 60 min. After irradiation, cells were kept in complete culture medium for an additional 48 h at 37°C in a 5% CO_2_ atmosphere. For cytotoxicity measurement, 10 μL Cell Titer reagent (Promega, Madison, WI, USA) was added to each well and incubated for 2 h. The absorption was recorded at 570 nm and 600 nm using a plate reader, respectively. The percentage of cell viability was determined by comparing treated cells with the untreated control.

## Results and Discussion

### Synthesis and Characterization of Co-drug (RB and PTX)-loaded Delivery Platform

**Figure 2:**
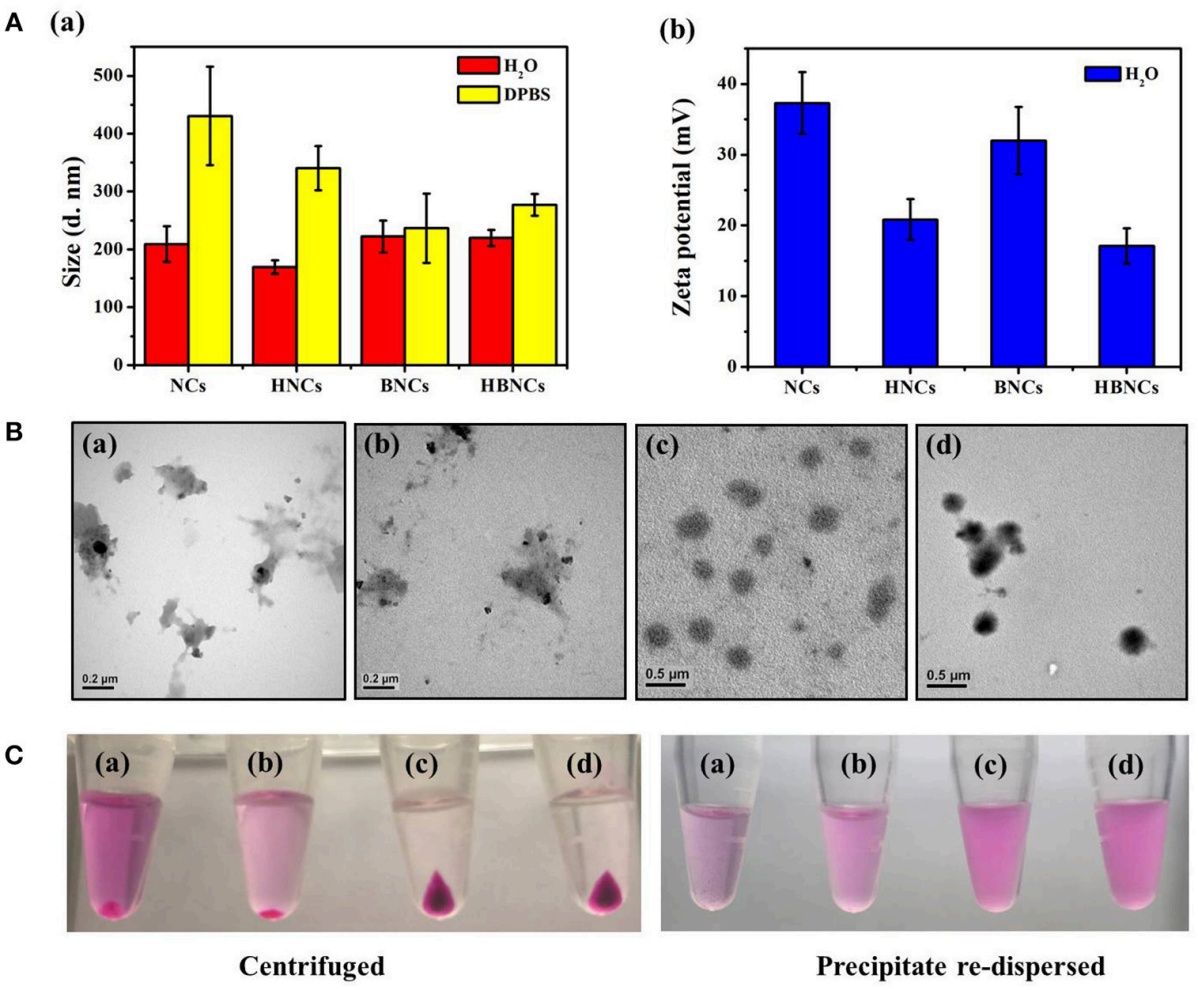
**(A)** Hydrodynamic size distributions and zeta potentials, **(B)** TEM images and **(C)** digital images of (a) NCs, (b) HNCs, (c) BNCs, and (d) HBNCs, respectively. The RB and PTX concentration added to each sample was fixed at 32 μM and 20 μM, respectively.

The size distributions of the resulting NCs, HNCs, BNCs, and HBNCs were characterized by DLS analysis. As depicted in Figure 2A, the average hydrodynamic size was found to be approximately 200 nm in ddH_2_O, while that of NCs showed the least uniform and polydispersed size distribution. The detection of a less positive value of the zeta potentials in HNCs and HBNCs than that of NCs and BNCs also suggests a successful surface grafting of HA via electrostatic interactions. Upon exposure to a buffer of high salinity (i.e., DPBS), a more pronounced increase of the aggregate size was observed for NCs (430.6 ± 84.9 nm) and HNCs (340.4 ± 38.2 nm) as compared to BNCs (236.7 ± 59.80 nm) and HBNCs (277.0 ± 18.7 nm), respectively. This finding suggests that the incorporation of BSA molecules into the cationic polymer nanoblends may strengthen the entangled network by the inclusion of numerous neighboring contacts (Dubois and Lavignac, 2014). TEM studies on particle morphology (Figure 2B) indeed show that NCs and HNCs exhibited a flaky and loosely packed structure, whereas BNCs and HBNCs possess a sphere-like compact structure. Next, the nanoparticles of different constitution were subjected to centrifugation and visualized by digital photographs in Figure 2C. Both of the BNCs and HBNCs presented the greatest quantity in the precipitate, indicating that nanoparticles made with BSA attain the highest production yield. The observation of an intense purple color (RB molecules) of the corresponding redispersion also suggests an improved formulation yield along with an enhanced cargo encapsulation efficiency. It is worthy to note that HNCs, as compared with NCs, was also identified to promote the mass production of colloidal nanoparticles. Therefore, the introduction of macroions of opposite charges (e.g., BSA or HA) has proved successful in condensation of flexible polyelectrolytes via multivalent counterion attractions. As expected, BNCs and HBNCs were found to be more stable in serum (10% FBS) containing culture medium than NCs and BNCs (Figure S1). No apparent sizes changes were observed for BNCs (214.2 ± 84.9 nm) and HBNCs (209.2 ± 34.3 nm) as compared to NCs (250.8 ± 103.6 nm) and HNCs (304.4 ± 93.5 nm), despite the fact that the surface adsorption of serum proteins may greatly improve the colloidal dispersibility in complex DMEM.

The optimal polymer constitute of the developed nanocarriers was systematically investigated based on the physicochemical properties and drug encapsulation efficiency (EE%). As shown in Table 1, sample (**a**) displayed a uniform size distribution of 220 ± 14 nm, a zeta potential of 17.1 ± 2.5 mV and RB loading efficiency of 60.7 ± 2.7%, indicating HBNCs are indeed an effective drug delivery nanoplatform. The nanocarriers without CTS (sample **b**) were also prepared with an elevated concentration of *b*PEI equal to that of CTS in sample **(a**) to ensure successful emulsification. Although sample (**b)** presents an acceptable hydrodynamic size, a remarkable decrease in RB loading (EE % = 23.2 ± 4.2%) was found. This is in accordance with the observation of a significant decline in zeta potential (1.8 ± 1.3 mV), revealing the essential structural function of CTS for skeletal network construction. PVA, a hydrophilic and water-soluble polymer, was chosen as a co-emulsifier to enhance the dispersion capability of the nanocarriers. As expected, nanocarriers prepared with a reduced amount of PVA (taken as 10 times lower) result in large hydrodynamic size (870 ± 114 nm in sample **(c)**). For nanocarriers lacking *b*PEI (sample **(d)**), a relatively low RB loading (33.1 ± 0.3%) was obtained. This result is consistent with the previous findings (Yeh et al., 2018), suggesting that *b*PEI with high charge densities (pK_a_ = 7.4–8.5) is effective for RB entrapment especially at physiological pH. Afterward, as described above, the positive value of zeta potential becomes less obvious after subjecting the nanocarrier to passivation with HA (Figure 2A). However, a twofold increase in the concentration of HA (sample **e**) leads no further change in zeta potential, but an increasing particle size (516 ± 112 nm); HA at a concentration of 0.2 mg/mL was chosen for further studies.

### Dual Drugs Loading

**Figure 3:**
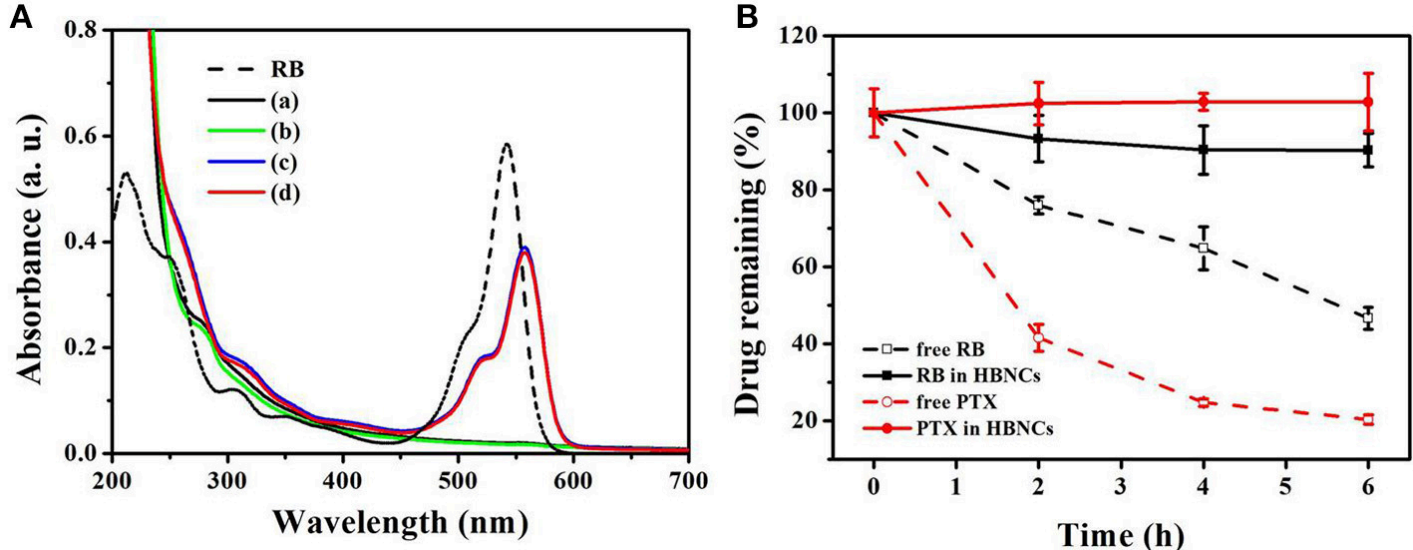
**(A)** UV-Vis absorption spectra of free RB, (a) HBNCs, (b) PTX-HBNCs, (c) RB-HBNCs, and (d) RB/PTX-HBNCs. **(B)** The leakage of drug molecules from RB/PTX-loaded HBNCs in DPBS (10% FBS). Each sample was dialysis against DPBS for 0–6 h. The amount of drug remaining inside the dialysis tubing was quantified using a calibration curve.

Next, the encapsulation of RB and PTX into HBNCs was characterized by ultraviolet-visible (UV-Vis) spectra in Figure 3A. The maximum absorption of RB is located at 530 nm (dash line). The UV-Vis spectrum of payload-free HBNCs **(a)** shows no characteristic absorption bands in the visible region, while a gradual rise in absorption toward shorter wavelengths, indicating the existence of colloidal structures. The subsequent loading of PTX leads to negligible changes in the UV-Vis absorption of HBNCs **(b)**. Whereas, an obvious peak at 560 nm was observed for RB-loaded HBNCs **(c)**. The red shift related to RB molecules after entrapment, also confirms the contribution of electrostatic effects originating from the surrounding environment (Uppal et al., 2011). No apparent change was observed for co-drug loaded HBNCs **(d)** as compared to RB-loaded HBNCs **(c)**. As no characteristic absorption peak of PTX had been observed in the UV-Vis spectra, HPLC was used for further identification. The encapsulation efficiency (EE, %) of RB and PTX into NCs, HNCs, BNCs, and HBNCs was also compared, respectively. As displayed in Table 2, a more pronounced drug loading was revealed for BNCs and HBNCs (EE_RB_% = c.a. 61%; EE_PTX_% = c.a. 54%) as compared with its non-BSA counterpart (EE_RB_% < 50%; EE_PTX_% < 45%). The introduction of HA to NCs also displayed an enhanced drug loading. Based on the fact of the abovementioned results (Figure 2C), the extent of drug entrapment is well-correlated with the recovery efficacy of the as-prepared nanoparticles.

**Table 2:** Drug encapsulation efficiency of different nanocarriers.

**Sample**	**EE_**RB**_ (%)**	**EE_**PTX**_ (%)**
NCs	40.8 ± 4.9	n.d.
HNCs	49.8 ± 1.4	44.7 ± 6.9
BNCs	60.4 ± 3.3	53.2 ± 7.9
HBNCs	60.7 ± 2.7	55.2 ± 8.9

*n.d., not determined*.

Premature drug leakage from co-drug loaded HBNCs was further confirmed by dialysis method. RB/PTX-loaded HBNCs dispersed in DPBS containing 10% FBS was dialyzed against DPBS at ambient temperature. Concurrently, free RB and PTX were also processed under the same condition as the control, respectively. As shown in Figure 3B, a negligible drug leakage (< 10%) was observed for RB/PTX-loaded HBNCs in 6 h under vigorous stirring, whereas the quantity of free drugs remaining inside the dialysis tubing continued to decline during the same period. This result implies that HBNCs was capable of reducing the non-specific drug action in complex biological conditions until triggered.

### Photo-Activated ROS-Responsive Drug Release

**Figure 4:**
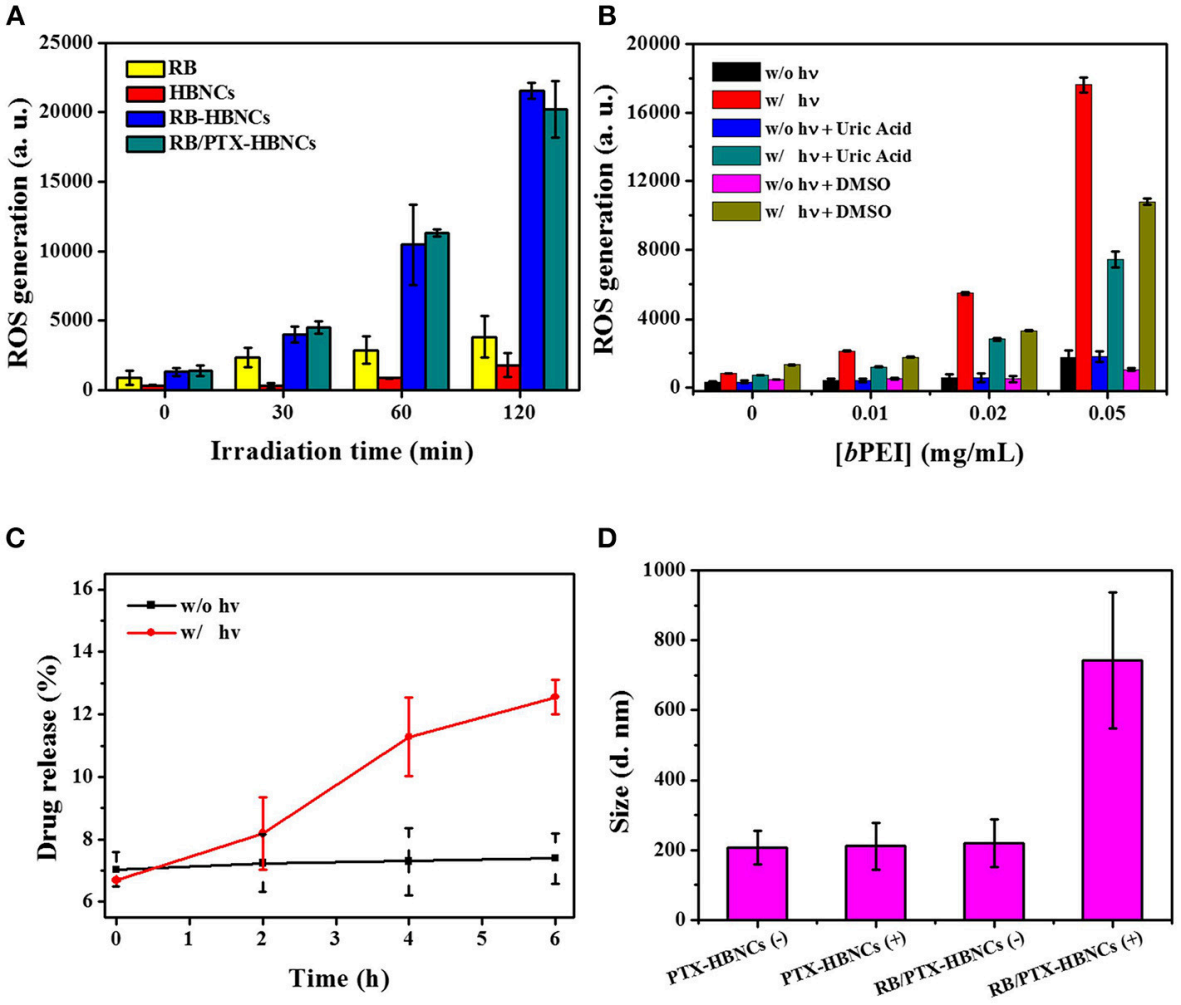
**(A)** ROS generation analyses of drug-loaded HBNCs in DPBS. DCFH-DA (5 μM) was added to each drug suspension, followed by a red light irradiation (632 nm, 15 mW/cm^2^) for 0–120 min. The concentration of RB was fixed at 40 μM. **(B)** ROS generation of RB in the presence of a serial concentration of *b*PEI triggered by 20-min light irradiation. **(C)** Light-induced RB release from co-drug loaded HBNCs in DPBS (10% FBS). An aliquot of sample was exposed to a red light for 0–6 h and the drug released at different time point was collected and measured by centrifugation at 40,000 g for 20 min at 4°C. **(D)** Hydrodynamic size changes of PTX-HBNCs and RB/PTX-HBNCs in DPBS (1% BSA) followed by light exposure (+) or darkness (–) of 6 h.

In order to investigate the photoresponsive characteristics of the developed nanoplatform, RB/PTX-HBNCs in DPBS were subjected to light illumination with their energy transmitted through a red filter with a center wavelength of 632 nm. ROS generation was studied using an oxidation-sensitive fluorescent probe, 2′,7′-dichlorodihydrofluorescein diacetate (DCFH-DA). As shown in Figure 4A, the relative ROS level of free RB under light illumination retained low contents along with the increase in irradiation time. Whereas, RB-HBNCs and RB/PTX-HBNCs produced a significantly increased amount of ROS vs. their cargo-free counterpart upon light exposure. This result may be attributed to the coappearance of type I photosensitization reactions in the presence of tertiary amines (Pan et al., 2011), leading to an enhanced ROS generation. As expected, a considerable increase in ROS production was observed in subjects with elevated *b*PEI concentrations (Figure 4B). Upon the respective addition of DMSO (hydroxyl radical scavenger) and uric acid (peroxynitrite scavenger), DCF fluorescence declined significantly with samples consisting of *b*PEI than that of free RB, further suggesting the involvement of additional ROS species. On the other hand, the photosensitization processes of RB in different formulations have also been probed by electron paramagnetic resonance (EPR) spectroscopy. As shown in Figure S2A, EPR spectra revealed the formation of TEMP-^1^O_2_ adducts (type II photosensitization) in all three RB samples, followed by light irradiation. However, only the samples equipped with *b*PEI (Figure S2B) allowed for the detection of type I products: DMPO-OOH (*a*_N_ = 14.2 G, $a_{\text{H}}^{\text{β}}$ = 11.4 G, and $a_{\text{H}}^{\text{γ}1}$ = 1.2 G) and DMPO-OH adducts (*a*_N_ = $a_{\text{H}}^{\text{β}}$ = 14.9 G) (type I photosensitization) (Janzen et al., 1987; Zhao et al., 2001). These results further confirm that RB-HBNCs can act as dual type I/II photosensitizer for improved photodynamic action under light exposure.

Next, the on-demand drug release behavior was investigated for the co-drug loaded HBNCs in responsive to the light-induced photosensitization process. As exhibited in Figure 4C, the cumulative release of RB was gradually increased over an extended irradiation period, as the non-irradiated counterpart remained almost at the original level. This result is consistent with our previous finding (Yeh et al., 2018), showing a specific RB release of HBNCs is remotely controlled. The observation of a blue shift in the spectrum feature of RB also confirmed an appreciable decrease in the ionic strength within the network of RB/PTX-HBNCs upon light illumination (Figure S3). Moreover, the irradiated nanocarriers also allowed a concurrent release of additional guest cargos; an approximately 30% PTX release was detected from RB/PTX-HBNCs after 6 h of light exposure, whereas the drug leakage was negligible in the dark. It is worth noting that the colloidal stability of RB/PTX-HBNCs dramatically decreased and hence, *in situ* coagulation was found during the laser irradiation process., A remarkable increase in hydrodynamic size was detected for RB/PTX-HBNCs vs. PTX-HBNCs followed by light irradiation (Figure 4D), further suggesting the active role of RB in mediating the light activated ROS generation for structural destability and the subsequent payload release.

### CD44-Targeted Cellular Uptake

**Figure 5:**
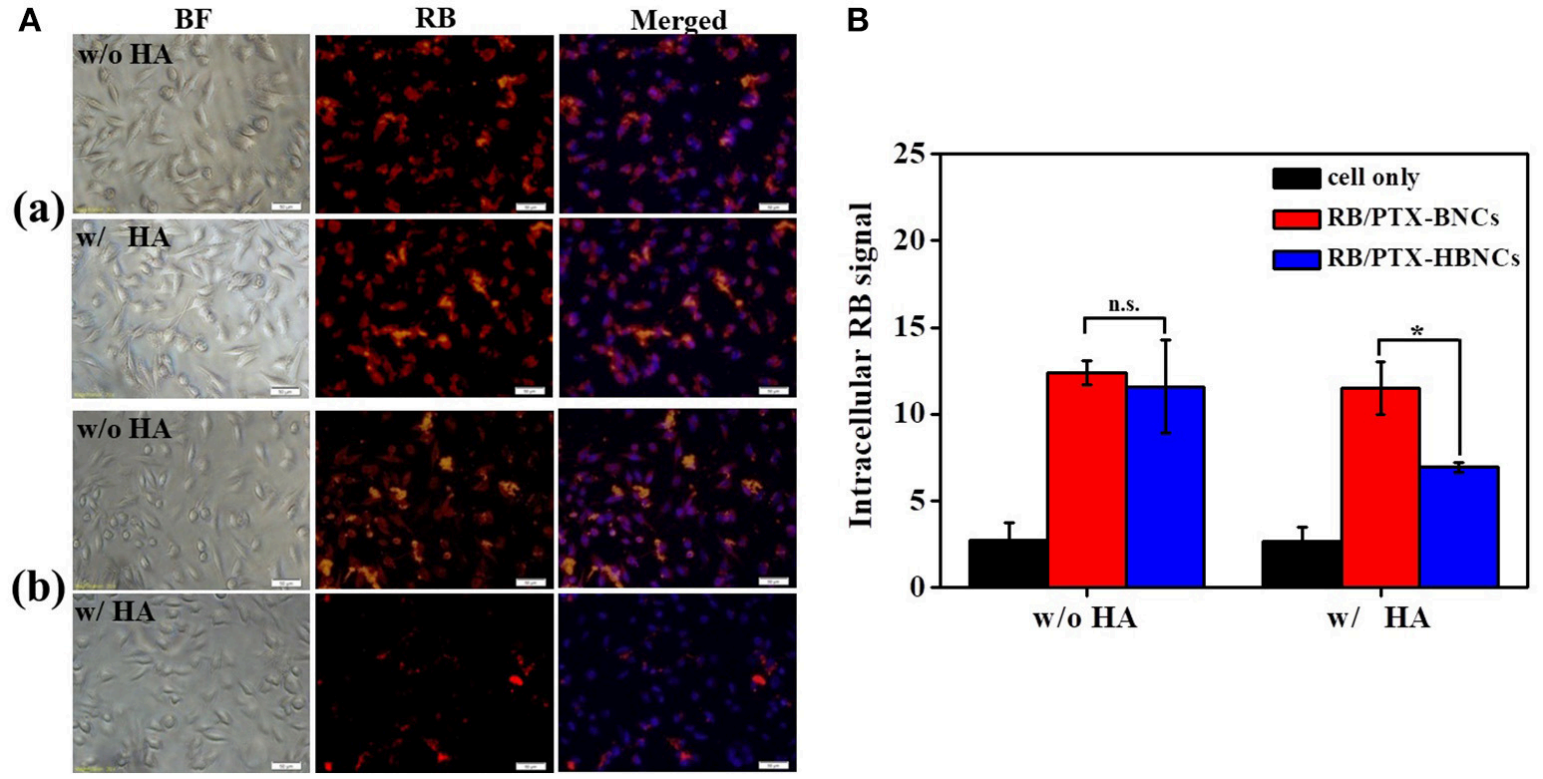
For the competitive binding studies, free HA was employed as a competitor. Both of the (a) (RB/PTX-BNCs)- and (b) (RB/PTX-HBNCs)-treated cells were co-incubated with or without free HA (10 mg/mL) in culture medium for 4 h. Cellular uptake of RB/PTX-HBNCs was analyzed by using **(A)** microscopy and **(B)** flow cytometry. Scale bar: 50 μm. Statistical significance at a level of *p < 0.05.

After the detailed characterization of physiochemical properties of the dual drug-loaded HBNCs, their interaction with prostate cancer Tramp-C1 cells was investigated using fluorescence microscopy. As shown in Figure S4, an obvious RB signal was detected in cells treated with RB-HBNCs and RB/PTX-HBNCs as compared with those exposed to free RB and PTX-HBNCs, respectively. This finding suggests that HBNCs offered a superior cellular uptake efficiency than that of free drugs. Moreover, competitive binding assays of HBNCs targeting Tramp-C1 cells were also performed using excess HA (Figure 5). The fluorescence signal from cells treated with RB/PTX-HBNCs decreased dramatically when they were co-incubated with free HA (*p* < 0.5). Whereas, (RB/PTX-BNCs)-exposed cells exhibited a negligible change in fluorescence (p > 0.5). It is considered that the cellular uptake of HBNCs by Tramp-C1 cells was accomplished by the specific recognition of HA with CD44 (Luo et al., 2000), showing great promise in targeted drug delivery. By contrast, BNCs without HA-surface grafting provided a strong positive charge (32.0 ± 4.8 mV in Figure 2A-b). It may eventually lead to undesirable side effects due to a high extent of non-specific cell interactions.

### Intracellular ROS Generation and Drug Delivery

**Figure 6:**
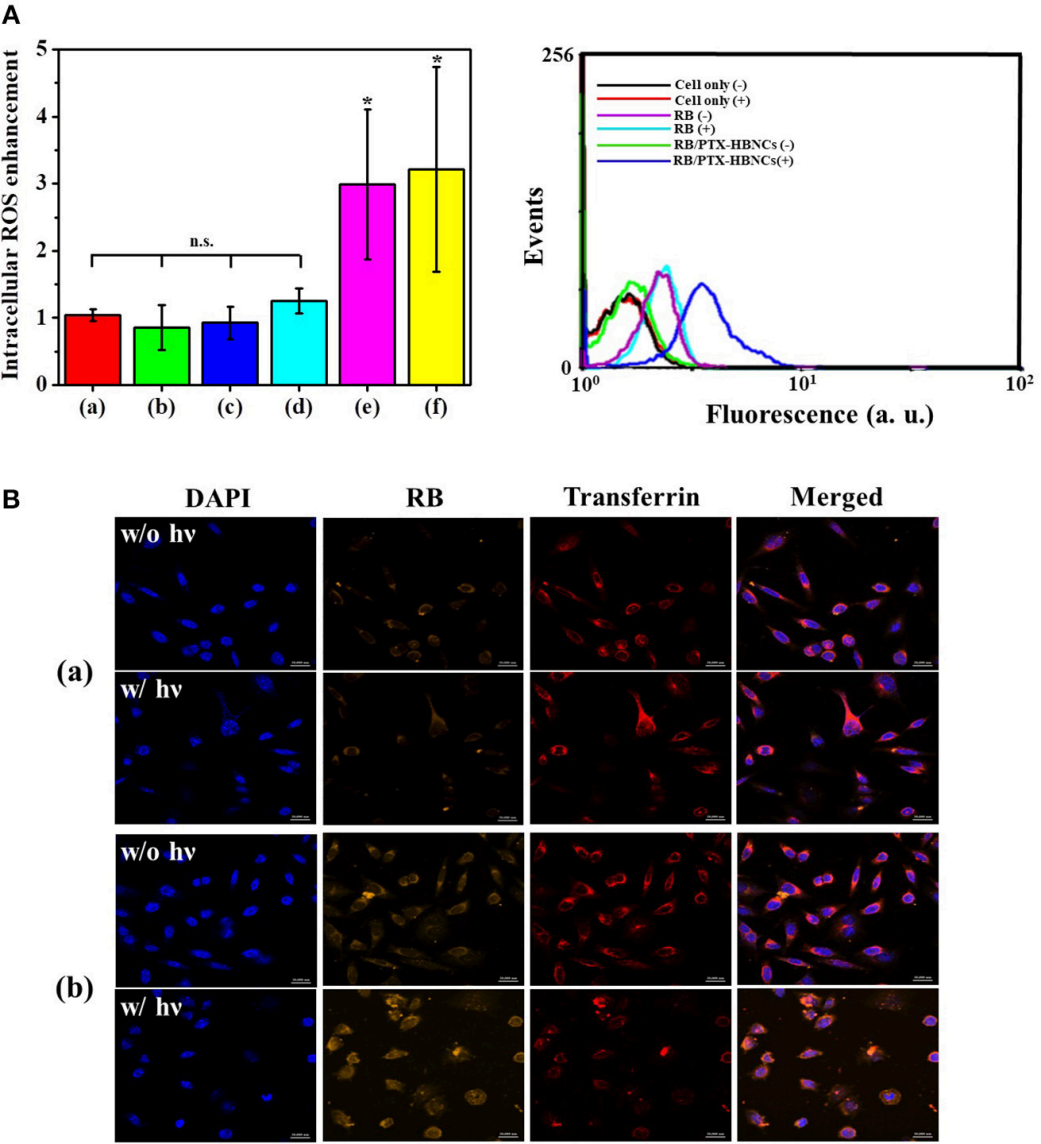
**(A)** Flow-cytometric analyses of intracellular ROS generation within Tramp-C1 cells of different treatment. The levels of intracellular ROS were determined using DCFH-DA (5 μM). **(Left)** The elevated cytosolic ROS (F/F_0_) of (a) non-treated cells, and cells incubated with (b) RB, (c) HBNCs, (d) PTX-HBNCs, (e) RB-HBNCs, (f) RB/PTX-HBNCs, respectively. The DCF signal of irradiated cells (F) was compared to that kept in darkness (F_0_). **(Right)** Representative histograms of non-treated cells, RB-treated cells and (RB/PTX-HBNCs)-treated cells in response to a red light irradiation (632 nm, 15 mW/cm^2^) for 1 h. **(B)** Confocal microscopic images of Tramp-C1 cells treated with **(a)** RB and **(b)** RB/PTX-HBNCs, respectively. RB concentration was fixed at 20 μM. After incubation in culture medium (10% FBS) for 6 h, cells were exposed to a red light for 1 h. Scale bar: 20 μm. n.s. > 0.05 and *p < 0.5 vs. non-treated cells.

Next, the intracellular PDT action mediated by RB/PTX-HBNCs was studied using DCFH-DA. As shown in Figure 6A, after 1 h of irradiation, the relative ROS level in cells treated with RB-HBNCs (column **e**) and RB/PTX-HBNCs (column **f**) was significantly increased (*p* < 0.5). Conversely, no significant difference in ROS production was observed for cells exposed to their RB-free counterparts (column **c** and **d**, respectively). RB-treated cells (column **b**) also exhibited negligible fluorescence changes after light illumination. Collectively, it can be concluded that HBNCs displayed both enhanced cellular uptake and improved PDT performance. A detailed study on the intracellular payload release and drug distribution was further investigated using confocal microscopy (Figure 6B). After 6 h of incubation, a distinct RB fluorescence was observed within the endolysosomal compartment, colocalized with fluorescent transferrin (transferrin-Alexa633) in the (RB/PTX-HBNCs)-treated cells. When exposed to the red light, the fluorescence emitted from RB molecules was significantly increased and spread diffusely. By contrast, negligible RB fluorescence changes were observed for cells treated with free RB under the same irradiation condition. This result confirmed that HBNCs which served to deliver dual drugs to the targeted cancer cells are effective for controlled payload release in response to the photodynamic reaction.

### *In vitro* Combination Therapy

**Figure 7:**
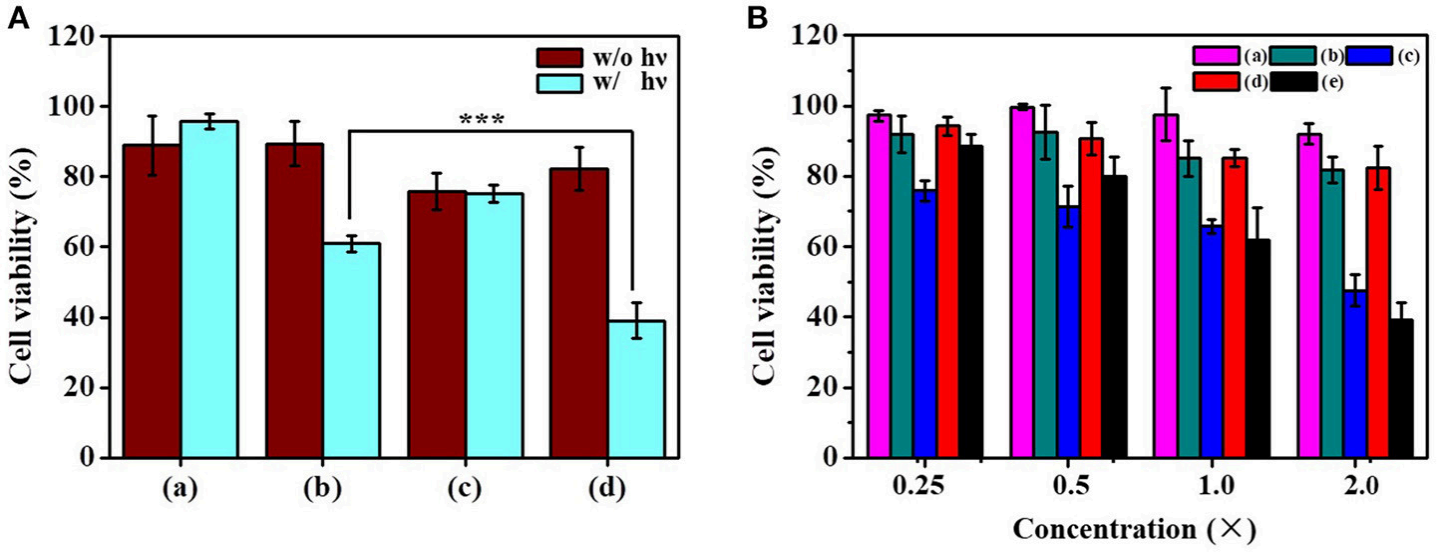
Evaluation of the cytotoxicity of dual-functional drug carriers toward Tramp-C1. **(A)** Cells were incubated with (a) HBNCs, (b) RB-HBNCs, (c) PTX-HBNCs, and (d) RB/PTX-HBNCs in DMEM (10 % FBS) for 6 h, followed by a red light irradiation (1 h). The concentration of RB and PTX was fixed at 40 and 20 μM, respectively. **(B)** Viability of cells treated with serial concentrations of (a) RB (–), (b) RB (+), (c) PTX (–), (d) RB/PTX-HBNCs (–), and (e) RB/PTX-HBNCs (+), respectively. After drug treatment, the survival of cells followed by light exposure (+) was compared to that kept in darkness (–). 1 × of RB and PTX represents 20 μM and 10 μM, respectively. Statistical significance at a level of ***p < 0.001.

To assess the anticancer activity of the dual functional drug carrier, Tramp-C1 cells were treated with RB/PTX-HBNCs and subjected to MTT assay for cell viability determination. As shown in Figure 7A, a minute toxic effect (< 18 ± 2%) was found in treated cells kept in the dark. However, a dramatic increase in cell death up to 61 ± 5% was observed when exposed to red light. This finding confirms the involvement of a phototoxic event that can be activated by an external trigger. In addition, the cytotoxic effects of RB-HBNCs and PTX-HBNCs were also assessed individually at equal drug concentration. PTX-HBNCs induced an approximately 24% decrease of cell viability while showing no response to photostimulation. In contrast, a viability reduction was detected, but to a lesser extent in (RB-HBNCs)-treated cells after light exposure. This finding indicates that the current approach is promising for on-demand dual-payload release, enabling simultaneous photodynamic and chemo actions for successful eradication of cancer cells. No cellular damage had been observed for cells treated with payload-free HBNCs, further suggesting a good biocompatibility of the developed nanocarrier. Moreover, the relative viability of cells incubated with free RB and light treatment was also examined. It was observed that <18 ± 6% of Tramp-C1 cells were killed at the maximum concentration tested (Figure 7B). This result correlates well with the previous finding (Yeh et al., 2018), showing that free RB is cell impermeant and thus of limited use *in vitro*. As for free PTX, an appreciable decrease in cell viability was detected in treated cells, owing to an adequate availability of small hydrophobic drugs toward cancer cells. However, PTX lacks sufficient tumor selectivity may potentiate the adverse side effects, leading to unwanted outcomes especially at high medication doses (Untch et al., 2016; Li et al., 2017). As compared to free drugs, RB/PTX-HBNCs showed a sufficient efficacy but a photo-triggerable anticancer activity is highly promising for targeted delivery of combined treatment in modern tumor therapy.

## Conclusions

We successfully developed a new ROS responsive drug delivery platform based on tripolymer mixtures (CTS, *b*PEI, PVA) for co-delivering photodynamic drugs and chemotherapeutics, which effectively combines both PDT and chemotherapy to improve the therapeutic efficacy in tumor cells. BSA was used to condense cationic tripolymer mixtures into stable nanocarriers. This drug carrier can also be readily functionalized with targeting moieties (HA, CD44 receptor), not only for enhancing the specific internalization by tumor cells, but also lowering down carrier's zeta potential for suitable application in biological systems. Notably, the designed ROS-responsive nanocarrier appeared to on-demand dual drugs release and delivery. Therefore, an improved therapeutic efficacy has been successfully demonstrated *in vitro* through combination therapy. With the advantages of easy synthesis, good biocompatibility, high specific binding affinity, and controllable capability of drug release, this nanocarrier will facilitate the development of next-generation cancer therapy in the future.

## Author Contributions

Y-CC design and perform experiments. AdV complete the experiments. H-PY synthesis of nanocarriers. YH write the paper. Y-FH make important changes to the paper and approve the final paper to be published.

### Conflict of Interest Statement

The authors declare that the research was conducted in the absence of any commercial or financial relationships that could be construed as a potential conflict of interest.
