# Supplementary material for: Development of Photo-Activated ROS-Responsive Nanoplatform as a Dual-Functional Drug Carrier in Combinational Chemo-Photodynamic Therapy
Source: Front Chem. 2019 Jan 9;6:647. doi: 10.3389/fchem.2018.00647 (PMC6334191; doi:10.3389/fchem.2018.00647)
Supplement: Supplementary file 1 [file Data_Sheet_1.docx]

Development of photo-activated ROS-responsive nanoplatform as a dual-functional drug carrier in combinational chemo-photodynamic therapy

Yu-Cheng Chang^a^, Andrea C. del Valle^a^, Huan-Pu Yeh^a^, Yue He^b,*^, and Yu-Fen Huang^a,^*

^a^Department of Biomedical Engineering and Environmental Sciences, National Tsing Hua University, Hsinchu, Taiwan, ROC

^b^Laboratory of Quality & Safety Risk Assessment for Citrus Products (Chongqing), Ministry of Agriculture, Citrus Research Institute, Southwest University, Chongqing,400712, China

**Supporting Information**

**Supplementary Scheme:**

**
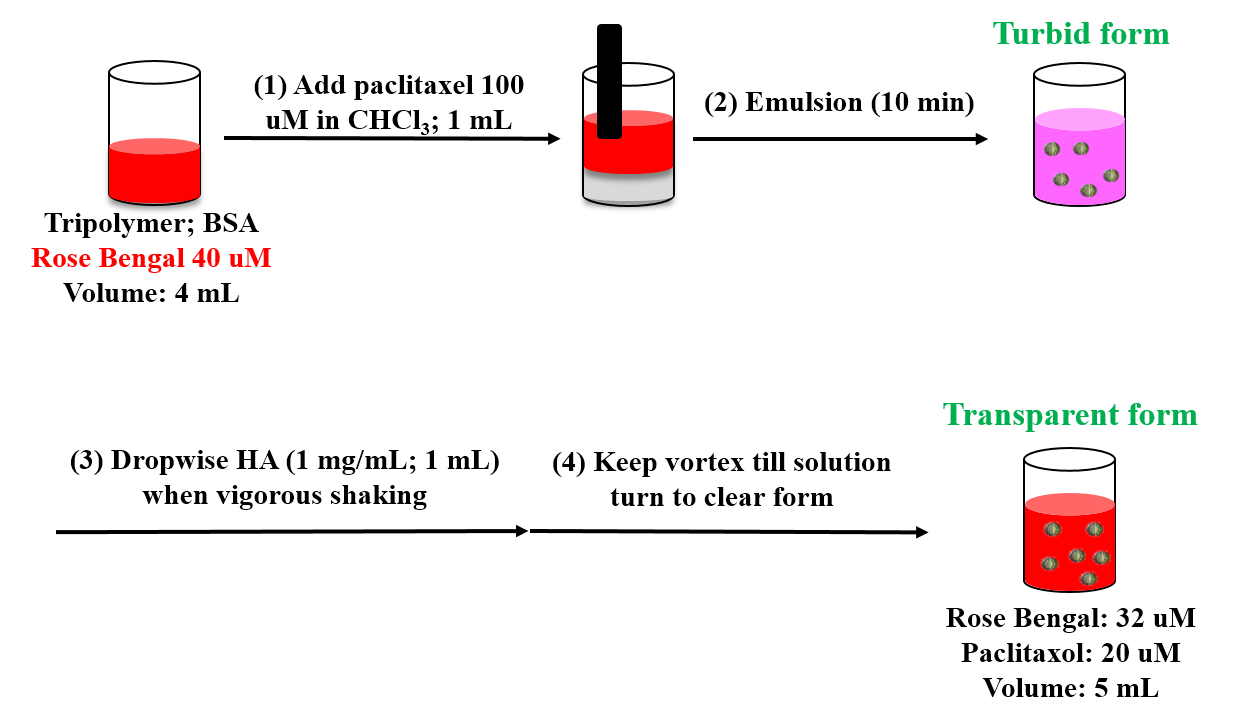
**

Scheme S1 Schematic illustration of detail information for fabrication of ROS-responsive polymeric nanocarriers as co-drug (RB and PTX) -loaded delivery platform through an oil-in-water emulsion method.

**Supplementary Table:**

**Supplementary Figures:**

**
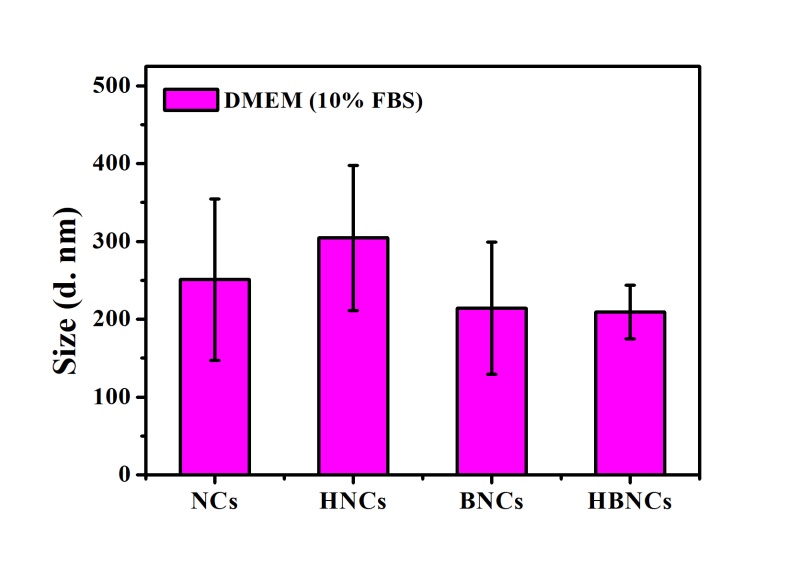
**

Figure S1. Hydrodynamic size distributions of drug-loaded nanocarriers in DMEM (10% FBS).


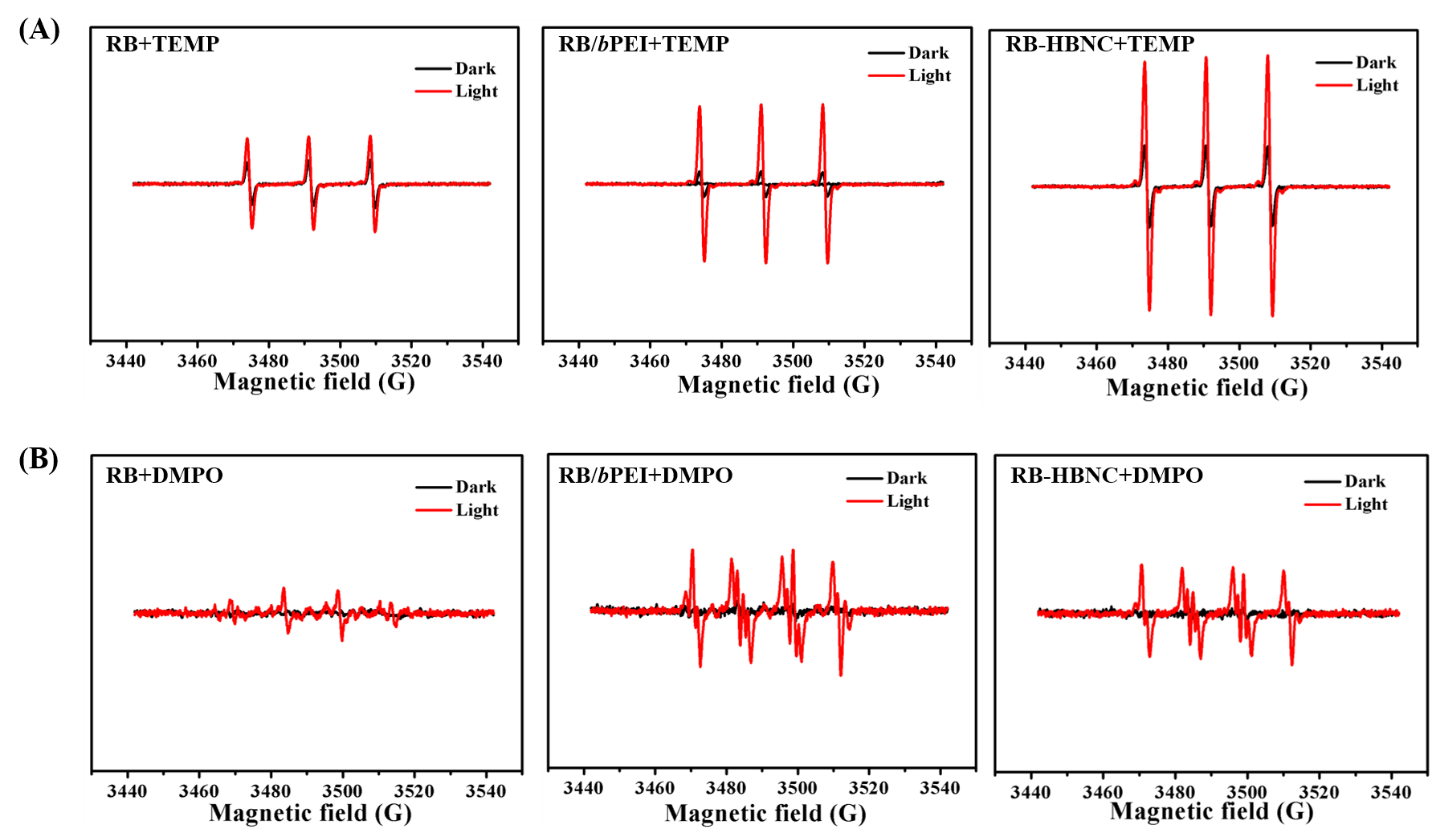


Figure S2. (A) Electron paramagnetic resonance (EPR) spectra of the TEMP adduct with singlet oxygen generated by RB, RB/bPEI, and RB-HBNC under 400 W high pressure mercury light (with a 480 nm long-pass filter) irradiation, respectively. (B) EPR spectra of the DMPO adduct with superoxide anion and hydroxyl radicals generated by RB, RB/bPEI, and RB-HBNC under red light irradiation, respectively.


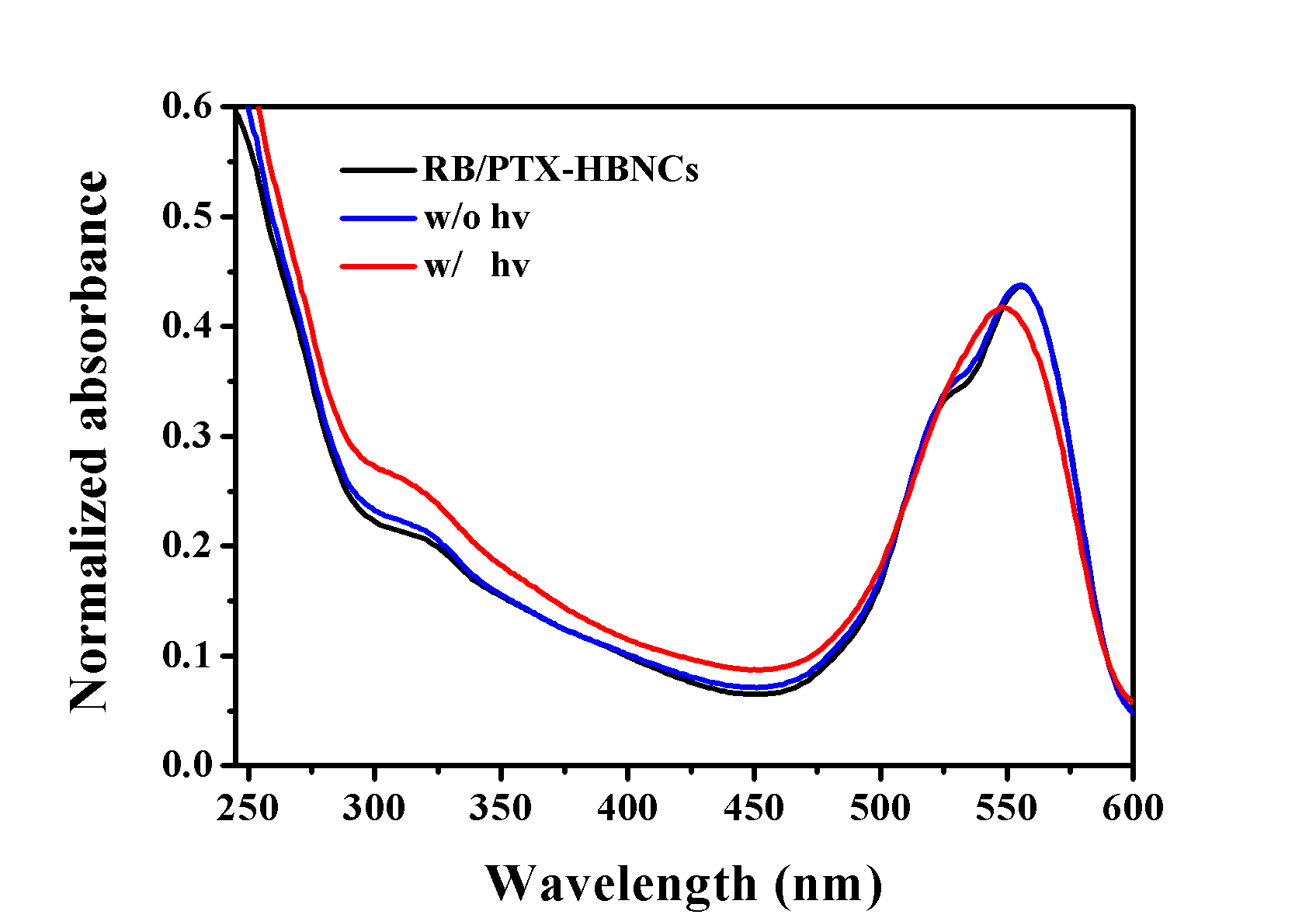


Figure S3. UV-vis spectra of RB/PTX-loaded HBNCs in DPBS with or without light exposure for 6 h.


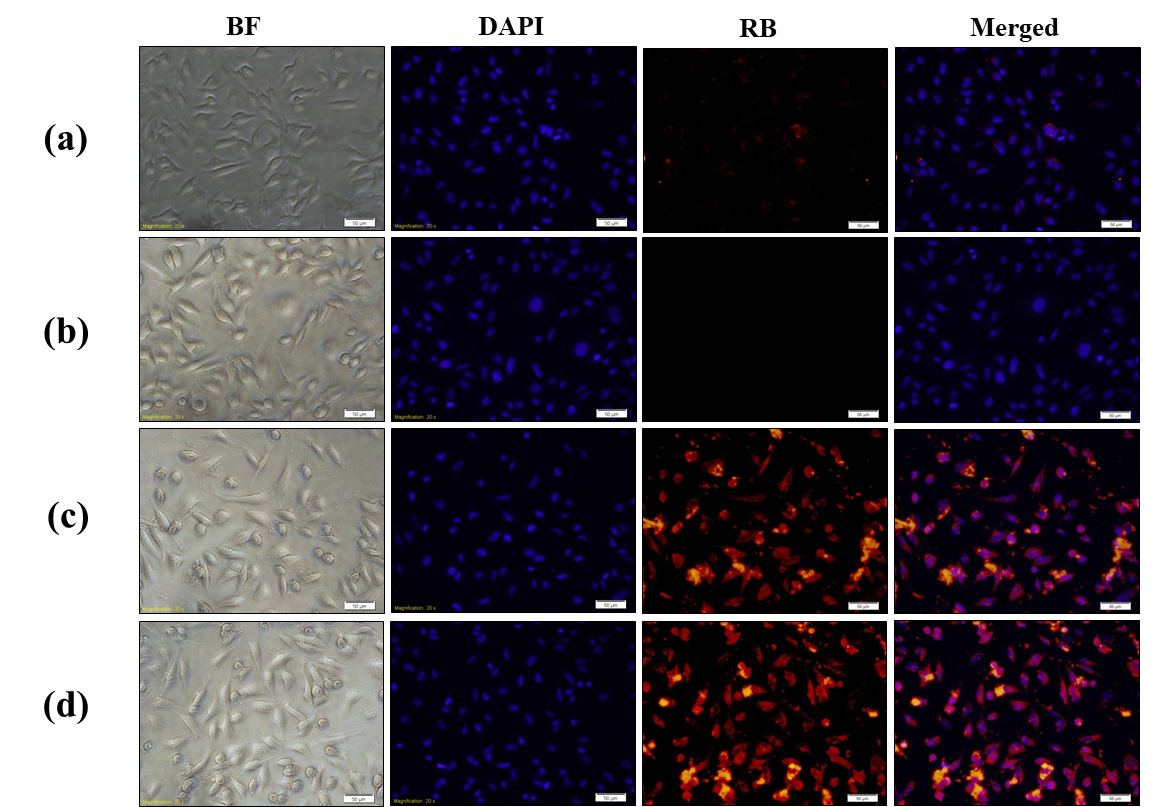


Figure S4. Microscopic images of Tramp-C1 cells with different treatment. Cells were incubated with (a) free RB, (b) PTX-HBNCs, (c) RB-HBNCs, and (d) RB/PTX-HBNCs in DMEM (10% FBS) for 6 h. Cells were then washed with DPBS, fixed with 4 % PFA for 15 min and stained with DAPI (1 µM) for 5 min before microscopic analyses.
